# Pancake Jumping of Sessile Droplets

**DOI:** 10.1002/advs.202103834

**Published:** 2022-01-14

**Authors:** Chenlu Qian, Fan Zhou, Ting Wang, Qiang Li, Dinghua Hu, Xuemei Chen, Zuankai Wang

**Affiliations:** ^1^ MIIT Key Laboratory of Thermal Control of Electronic Equipment School of Energy and Power Engineering Nanjing University of Science and Technology Nanjing 210094 China; ^2^ Department of Mechanical Engineering City University of Hong Kong Hong Kong 999077 China

**Keywords:** bending deformation, magnetic field actuation, pancake jumping, superhydrophobic magnetically responsive blades array

## Abstract

Rapid droplet shedding from surfaces is fundamentally interesting and important in numerous applications such as anti‐icing, anti‐fouling, dropwise condensation, and electricity generation. Recent efforts have demonstrated the complete rebound or pancake bouncing of impinging droplets by tuning the physicochemical properties of surfaces and applying external control, however, enabling sessile droplets to jump off surfaces in a bottom‐to‐up manner is challenging. Here, the rapid jumping of sessile droplets, even cold droplets, in a pancake shape is reported by engineering superhydrophobic magnetically responsive blades arrays. This largely unexplored droplet behavior, termed as pancake jumping, exhibits many advantages such as short interaction time and high energy conversion efficiency. The critical conditions for the occurrence of this new phenomenon are also identified. This work provides a new toolkit for the attainment of well‐controlled and active steering of both sessile and impacting droplets for a wide range of applications.

## Graphical abstract

A new jumping of sessile water droplets is discovered in a pancake shape, which is triggered by the fast response and quick deformation of the superhydrophobic magnetic blades array. The pancake jumping shows signatures of short interaction time and high energy conversion efficiency.

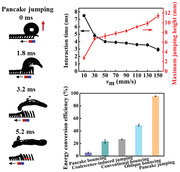

## 1 Introduction

Rapid droplet shedding from various surfaces has been extensively investigated over the past decade owing to its scientific importance and practical applications in anti‐icing,^[^ ^1^, ^2^ ^]^ droplet condensation,^[^ ^3^, ^4^ ^]^ self‐cleaning,^[^ ^5^ ^]^ and electricity generation.^[^ ^6^, ^7^ ^]^ In particular, on superhydrophobic textured surfaces, impinging droplets exhibit a complete rebound at the end of its retraction due to low friction,^[^ ^8^, ^9^, ^10^, ^11^, ^12^, ^13^, ^14^, ^15^, ^16^, ^17^ ^]^ or pancake bouncing at the maximum spreading.^[^ ^18^, ^19^, ^20^ ^]^ Pancake bouncing breaks the theoretical Rayleigh limit with an even low contact time of ≈3.4 ms, but the manifestation of such a phenomenon requires the elegant control of the macrotextures and impacting velocity of droplets (Section S1, Supporting Information).^[^ ^21^ ^]^

On the other hand, sessile droplets shedding from surfaces in a bottom‐to‐up manner is even more challenging. Coalescence‐induced droplet jumping occurs on superhydrophobic surfaces caused by the release of surface energy during the condensation process,^[^ ^22^, ^23^, ^24^, ^25^, ^26^ ^]^ but it is limited by the low energy transfer efficiency and thus suffers from a small jumping velocity.^[^ ^27^, ^28^ ^]^ The directional movement of sessile droplets can be achieved on responsive surfaces through constructing energy gradient, or resorting to stimuli such as temperature,^[^ ^29^, ^31^ ^]^ pressure,^[^ ^32^, ^33^, ^34^, ^35^ ^]^ optical,^[^ ^36^, ^37^ ^]^ electrical^[^ ^38^, ^39^ ^]^ or magnetic fields,^[^ ^40^, ^41^, ^42^, ^43^, ^44^, ^45^, ^46^ ^]^ etc. In particular, owing to its advantages of instantaneous response,^[^ ^42^ ^]^ low energy consumption, flexible/convenient/safe controllability, and good biocompatibility, magnetic actuation has emerged as a promising approach to manipulate droplet motion.^[^ ^43^ ^]^ However, the motion of sessile droplets normally occurs in the plane. One elegant approach for achieving out of plane motion or shedding lies in the spontaneous droplet trampolining under a low‐pressure environment, but is limited in practical application (Section S1, Supporting Information).^[^ ^32^ ^]^

Herein, we report on the discovery of a bottom‐to‐up jumping of sessile water droplets in a pancake shape that displays short interaction time and high energy conversion efficiency. Our method lies in designing the superhydrophobic magnetically responsive blades array (SMBA), which can be easily fabricated through the combined soft lithography and laser ablation technology. SMBA can effectively translate the droplet interfacial energy into kinetic energy to impel sessile droplets to pancake jump via the fast response of bending deformation of blades array under magnetic field actuation. We investigate the critical surface structural parameters and magnetic field properties for the manifestation of pancake jumping. We further demonstrate the rapid shedding of cold droplets on SMBA, which can be exploited for enhanced anti‐icing applications.

## 2 Results

### 2.1 SMBA Design and Fabrication

**Figure 1:**
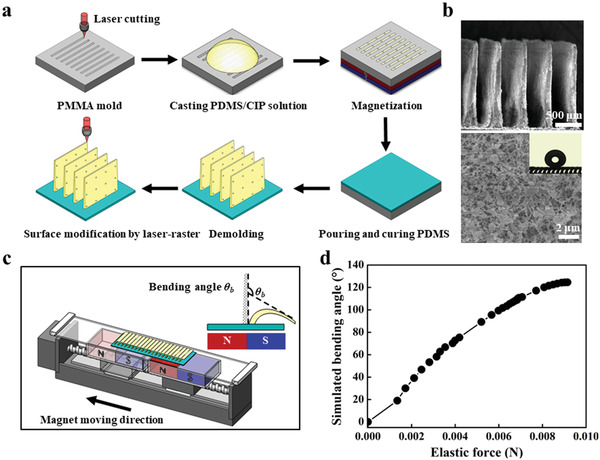
Fabrication and characterization of superhydrophobic magnetically responsive blades array (SMBA). a) Schematic drawing of the fabrication process of SMBA. b) SEM images of SMBA after laser ablation. The sidewall of the blades array is covered with nanoscale bumps after laser ablation. The local water contact angle on the bending SMBA is 160°. c) Schematic illustration of the bending deformation behavior of individual blades on SMBA in response to an external magnetic field generated from a magnet block. Bending angle *θ*_b_ is defined as the angle between the tangent line at the base of the blade and the tangent line at the top of the blade. d) The elastic force results from bending deformation of the blade.

The SMBA was made through the combined soft lithography and laser ablation technology (**Figure** **1**a). Briefly, a polymethylmethacrylate (PMMA) master mold patterned with rectangular hole arrays was first created using a CO_2_ laser cutter. Then, a PDMS/carbonyl iron powder (CIP) mixture with a weight ratio of 70% was cast into the PMMA mold. To induce alignment of the iron particles in the mold holes, a permanent Neodymium‐iron‐boron (NdFeB) magnet was placed underneath the mold to generate a vertical magnetic field. After wiping off excess PDMS/iron particle solution outside the holes with a razor blade, a pure PDMS solution was also poured onto the mold to serve as the substrate and to support the blades. After curing at 80 °C for 4 h and peeling off from the mold, we used laser‐raster scanning across the surface to form nanoscale bumps of ≈500 nm diameter and reduced surface energy via pyrolyzation of PDMS.

Figure 1b shows the structure of the as‐fabricated SMBA, where blades have an average height *h*, top width *w*_1_, bottom width *w*_2_ of ≈1675, 40, 200 µm, respectively, with an aspect ratio $\beta \;=\;h/(\frac{w_{1}+w_{2}}{2})$ of ≈14 (Figure S1, Supporting Information). The presence of nano‐scale bumps on SMBA amplifies the local hydrophobic property, endowing a global apparent water contact angle of ≈160°, contact angle hysteresis of ≈3°, and sliding angle of ≈2°. For comparison, we also prepared three sets of superhydrophobic magnetically responsive surfaces with aspect ratio *β* of ≈5, ≈8, and ≈11, respectively (Table S1, Supporting Information).

To examine whether SMBA can achieve a collective force sufficiently large to actuate the motion of droplets by the deformation of blades array, we first investigate the bending behavior of individual blades in response to an external magnetic field. The intrinsic magnetic field is generated from a magnet block made of two opposite square NdFeB magnets (45×45×20 mm), in which the largest magnetic field occurs in the junction of two opposite magnets. The response of magnetic blades can be tailored by moving the block horizontally underneath the SMBA (Figure 1c). Initially, when the magnet block is far away from the SMBA, blades are subject to a negligible magnetic flux density and maintain their original straight state. As the magnet block gradually approaches SMBA, the rightmost blade feels an increasing magnetic flux density and then bends in a direction toward the maximum magnetic field strength, i.e., the center of the magnetic block. We quantify the degree of deformation of blades using the bending angle *θ*_b_, defined as the angle between the tangent line at the base of the blades and the tangent line at the top of the blades. We found that the maximum bending angle *θ*_b_ is ≈125°, corresponding to a magnetic flux density *B*_0_ of ≈285 mT (Figure S2a, Supporting Information). Further horizontally moving the magnet block away to the left direction leads to a reduced magnetic flux density and a full recovery of the bending blade back to its initial straight state. Note that the bending‐to‐straight response behavior of the blade on SMBA is highly dependent on the magnet moving velocity, *v*_m_. A large *v*_m_ induces a rapid decrease in magnetic flux density, and subsequently a fast bending‐to‐straight response of the blade. For *v*_m_ ≈ 10 mm s^–1^, the bending angle of a single blade of SMBA is reduced from 125° to 82° within 2 ms and down to 0° (straight state) at 5.2 ms. In contrast, for *v*_m_ ≈ 150 mm s^–1^, the response time is decreased to 1.8 ms (Figure S2b, Supporting Information). These results suggest that the dynamic response of individual blades can be closely regulated by controlling the motion of the external magnet or the corresponding magnetic flux density.

We then numerically simulate the deflection of a single blade under external magnetic field actuation using the coupled Fluid‐Structure interaction method (Figure S3 and Section S2 in the Supporting Information). When a single blade is deposited on the magnet block, it's bending of the single blade results from the cooperation of magnetic force and elastic force. The magnetic force is generated from the gradient in external magnetic field strength, a quantity determined by the distance between the blade and the magnet (Figure S4, Supporting Information). Meanwhile, as shown in Figure 1d, the elastic force results from the deformation of the blade. Balancing the elastic force and the magnetic force (Figure S5, Supporting Information), the bending angle of a single blade can be simulated. The maximum bending angle is simulated to be ≈124.5°, in accordance with the experimental observation.

### 2.2 Pancake Jumping on SMBA

**Figure 2:**
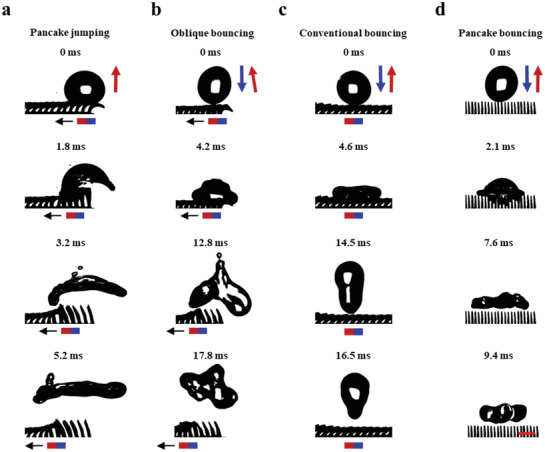
Droplet dynamics on SMBA. a) Selected snapshots showing a water droplet deposited on SMBA forms a large arc at 1.8 ms, jumps away from the surface at 3.2 ms, and forms a pancake shape in the air at 5.2 ms when *v*_m_ ≈ 150 mm s^–1^. b) Selected snapshots showing an impinging droplet (*We* ≈ 5) obliquely bounces off SMBA at 12.8 ms when *v*_m_ ≈ 150 mm s^–1^. c) Selected snapshots showing an impinging droplet (*We* ≈ 10) exhibits a conventional bouncing on SMBA which is above a stationary magnet and detaches from the surface at 14.5 ms. d) Selected snapshots showing an impinging droplet (*We* ≈ 34) bounces off SMBA in a pancake shape at 7.6 ms without magnetic field actuation. Scale bar: 1 mm.

Droplet dynamics on SMBA are deeply modified by the horizontal motion of the underlying magnet block. When the magnet block is directly placed below SMBA, corresponding to a maximum magnetic flux density *B*_0_ ≈ 285 mT, SMBA can be treated as the tilted blades array, on which a 10 µL deionized sessile water droplet sitting at the rightmost of the surface exhibits symmetric profile. As shown in **Figure** **2**a, under a horizontal motion of magnet at *v*_m_ ≈ 150 mm s^–1^ against the tilt direction, these tilted blades under the trigger of magnetic flux recover to the straight state within ≈ 1.8 ms. During this process, a restoring force is produced and transforms the initially symmetric profile of the droplet into an arc shape. The deforming droplet jumps off the underlying surface at 3.2 ms and further displays in a pancake shape in the air (Movie S1, Supporting Information). This unique bottom‐to‐up jumping of the sessile droplet, termed as pancake jumping, is different from the pancake bouncing of impinging droplet.^[^ ^18^ ^]^ Pancake jumping results from the fast and dynamic response of surfaces to the external trigger, in which different droplet behaviors are possible to be achieved based on one surface design without the need of initial kinetic energy input from droplets. In contrast, pancake bouncing is limited to impinging droplets alone, and also requires the elegant control of surface macrotextures and the initial kinetic energy of impinging droplets. However, the droplets sitting on a heated substrate^[^ ^29^ ^]^ or in a low‐pressure environment^[^ ^32^ ^]^ or on a vibrating substrate^[^ ^34^ ^]^ can only jump upward under a vertical upward force (Section S3, Supporting Information). It should be noted that there are still some limitations of pancake jumping: an external magnetic/elastic energy of the blades array is required, and the sessile droplet needs to be deposited on the rightmost of SMBA initially owing to the surface edge effect (Figure S7a and Section S4 in the Supporting Information).

Different droplet behaviors are also achieved on SMBA (Table S2 and Figure S8, Supporting Information). For a droplet impacting on the rightmost blades of SMBA with a Weber number (*We*) ≈ 5 (initial impacting height ≈ 6.3 mm), the droplet quickly spreads and bounces off the surface obliquely before reaching its maximum spreading, termed as oblique bouncing (Figure 2b and Movie S2, Supporting Information). Here, *We*  = *ρv* ^2^ *D*/*γ* , where *ρ* is the liquid density, *v* is the droplet impact velocity, *D* is the droplet diameter and *γ* is the liquid surface tension. In this process, the magnet moving velocity *v*_m_ is 150 mm s^–1^. Due to the surface edge effect, the droplet bouncing dynamic is strongly influenced by the position of where the droplet impacts (Figure S7b and Section S4 in the Supporting Information). For a stationary magnet, the impinging droplet exhibits conventional bouncing on the bending blades of SMBA due to the insufficient dynamic magnetic response of blades deformation. For instance, a droplet (*We* ≈ 10 and initial impacting height ≈ 11.5 mm) impacting on SMBA above a stationary magnet (the bending blades array forming an end‐to‐end continuous surface) retracts on the bending blades at 4.6 ms and completely detaches from SMBA at 14.5 ms (Figure 2c and Movie S3, Supporting Information). Besides, without the magnetic field actuation, the blades array exhibits in a straight state. The droplet impinging on SMBA (*We* ≈ 34 and initial impacting height ≈ 40 mm) deeply penetrates into the straight blades at 2.1 ms and bounces off SMBA at 7.6 ms in a pancake shape at the end of the maximum lateral extension (Figure 2d and Movie S4, Supporting Information). Such a phenomenon is consistent with the existing study.^[^ ^18^ ^]^ The Weber number for the onset of pancake bouncing ranges between 23 and 43.

### 2.3 Features of Pancake Jumping

**Figure 3:**
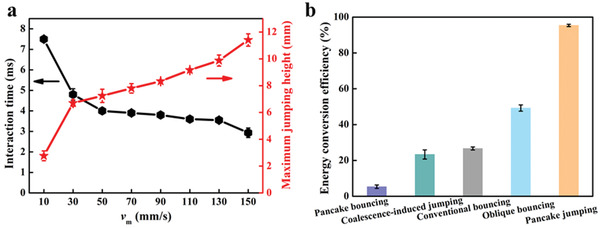
Signatures of pancake jumping. a) The droplet surface‐interaction time and droplet maximum jumping height for pancake jumping as a function of *v*_m_. The shortest interaction time of pancake jumping is 2.9 ms and the maximum jumping height of pancake jumping is 11.4 mm at *v*_m_ ≈ 150 mm s^–1^. b) Comparison of energy conversion efficiency *φ*, *ϕ*  = *E_k_*/*E_s_* , between different droplet behaviors. The *φ* of pancake jumping droplet at *v*_m_ ≈ 150 mm s^–1^ is ≈ 95%, larger than that of the pancake bouncing droplet (*φ* ≈ 5%), the oblique bouncing droplet (*φ* ≈ 49%), and the coalescence‐induced jumping droplet (*φ* ≈ 23%).

One interesting signature of droplet pancake jumping lies in its short interaction time, which is defined as the time interval that the droplet deforms on SMBA. As shown in **Figure** **3**a, the interaction time exhibits remarkable dependence on the magnet moving velocity *v*_m_. With the increase of *v*_m_, the interaction time ranges from 7.5 to 2.9 ms when *v*_m_ increases from 10 to 150 mm s^–1^. We further compared the maximum jumping height *h*_0_, which is defined as the height between the droplet centroid and the bending blades array (Figure S9, Supporting Information), of droplet pancake jumping at varied *v*_m_. The *h*_0_ of pancake jumping (≈ 11.4 mm) at *v*_m_ ≈ 150 mm s^–1^ is four‐fold larger than that of pancake jumping when *v*_m_ ≈ 10 mm s^–1^.

Pancake jumping of the droplet can be divided into two periods: droplet deforming when it contacts with SMBA; droplet jumping in the air when it is not in contact with SMBA. There exist three energy conversion mechanisms: magnetic/elastic‐to‐rotation energy within the blades array; rotation‐to‐interfacial energy between the blades array and the droplet; interfacial‐to‐kinetic energy within the droplet. When the droplet is in contact with SMBA, the droplet deformation is propelled by the bending‐to‐straight response behavior of the blades array (Figure S10, Supporting Information). During this process, the energy is converted from rotation energy *E*_rot_ of the blades array to droplet interfacial energy *E*_s_. Initially, the stored elastic energy of the bending blades array is equal to the magnetic energy. When the bending blades array recovers to the straight state under a horizontal motion of the magnet, the unbalance of magnetic torque *T*_m_ and elastic torque *T*_e_ induces *E*_rot_ of the blades array (Figure S11, Supporting Information). *E*_rot_ is related to magnetic energy and elastic energy. Based on the conservation of energy, *E*_rot_ is completely converted into *E*_s_ of the droplet. When the droplet jumps off the surface, the energy is converted from *E*_s_ to droplet kinetic energy *E*_k_. One part of the *E*_s_ is transformed into the *E*_k_ and the other part is dissipated by the adhesion energy between the droplet and the surface. This energy conversion process is characterized by the energy conversion efficiency *φ*. The energy conversion efficiency *φ* (*ϕ*  = *E_k_*/*E_s_* , defined as the ratio of the kinetic energy *E*_k_ to the interfacial energy *E*_s_ of pancake jumping droplet), which is equal to the ratio of droplet kinetic energy after and before droplet impinging on the surface.

Another intriguing finding of our pancake jumping is the high energy conversion efficiency of the droplet. As shown in Figure 3b, *φ* of droplet pancake jumping is 95% at *v*_m_ ≈ 150 mm s^–1^, ≈ 4 times larger than that of the coalescence‐induced condensate droplet jumping on the superhydrophobic surface (Figure S12, Supporting Information). This might be attributed to the effective and fast magnetic actuation exerted on the droplet, which provides significant *E*_s_ to impel it to jump from SMBA in a pancake shape. Whereas, the *φ* of oblique bouncing droplet is only ≈ 49% under the same condition. The small *φ* of the oblique bouncing droplet is owing to the coupling of downward impingement of droplet and upward blades array deformation, resulting in the dissipation of a part of *E*_s_ of the droplet by the bending‐to‐straight response behavior of the blades array. In addition, the *φ* (≈27%) of droplet conventional bouncing is far smaller than that of the pancake jumping droplet, due to the absence of *E*_s_ provided by bending blades deformation. The pancake bouncing droplet presents the smallest *φ* (≈5%) due to the reduction in *E*_s_, which is consumed by the existing air cushion between the straight blades array on SMBA.

Moreover, the *φ* of pancake jumping droplets in this work is larger than that of existing jumping droplets or bouncing droplets in the literatures (Table S3, Supporting Information). For example, the *φ* of pancake jumping droplets is 23 times larger than that of the droplet bouncing off the flexible superhydrophobic surface (≈4%).^[^ ^15^ ^]^ Taken together, our pancake jumping of droplet owns remarkable advantages of short interaction time and high energy conversion efficiency, which benefit for the rapid droplet shedding from the surface.

### 2.4 Design Principle

To predict the boundary conditions for the occurrence of pancake jumping, we develop a theoretical model that links structural parameters and magnetic field properties by considering droplet shape and energy transition (Section S5, Supporting Information). Assuming that a spherical‐cap droplet with radius *R* and static contact angle *θ_c_* is placed on bending blades, the droplet has a liquid‐vapor surface area *A_lv_* =  2*πR* ^2^(1 − *cosθ*_*c*_). When the bending blades recover to the original straight state, the liquid‐vapor surface area of the droplet increases to *A* *_lv_* ' (Figure S10, Supporting Information). During this process, the increase in the interfacial energy results from the droplet transformation and rotation, which is induced by the elastic torque in the bending blades and magnetic torque (Figure S11, Supporting Information). Based on the conservation of energy, we yield *A* *_lv_* ' as

(1) $$A_{lv}^{\prime }=\pi R^{2}\left(2-3\cos\theta_{c}+\mathrm{co}s^{3}\theta_{c}\right)+f_{1}\frac{{\theta_{b}}^{2}}{\beta }+f_{2}\frac{\left|cos\theta_{b}\right|}{{\left(v_{m}t_{m}\right)}^{3}}$$

where *E* ≈ 1.3 MPa is the elastic modulus of a blade,^[^ ^44^ ^]^ *V*_m_ is the volume of the carbonyl iron particles inside a magnetic blade, *M* is the magnetization of the carbonyl iron particles. $f_{1}=\frac{cEI}{\gamma (w_{1}+w_{2})}\;$ and $f_{2}=\frac{V_{m}MB_{0}}{\gamma }\;$, where *c* is the parametric angle coefficient and *I* is the second moment of inertia. To determine the extent of droplet interfacial energy variation during the bending‐to‐straight response process of the blades array, we nondimensional relevant parameters and characterize them as *k*, which is the ratio of the interfacial energy of droplet after and before the bending blades recovering to the straight state

(2) $$k\;=\frac{{A^{\prime }}_{lv}}{A_{lv\;}}∼\frac{{\theta_{b}}^{2}}{\beta }+\frac{\left|cos\theta_{b}\right|}{{\left(v_{m}t_{m}\right)}^{3}}+1$$

**Figure 4:**
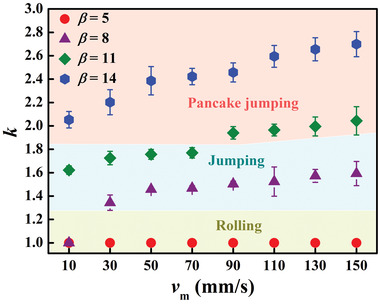
Design diagram of pancake jumping. The variation of droplet interfacial energy ratio $k\;={A_{lv}}^{\prime }/A_{lv\;}\;$as a function of *v*_m_ on SMBA featuring with various *β*. The increase in *v*_m_ or *β* results in a significant increase in kinetic energy to impel the droplet to jump. The droplet pancake jumping occurs on SMBA with *β* ≈ 11 when *v*_m_ ≥ 90 mm s^–1^ (*k* ≥ 1.8) or on SMBA with *β* ≈ 14 when *v*_m_ ≥ 10 mm s^–1^ (*k* ≥ 2.1).

**Figure 5:**
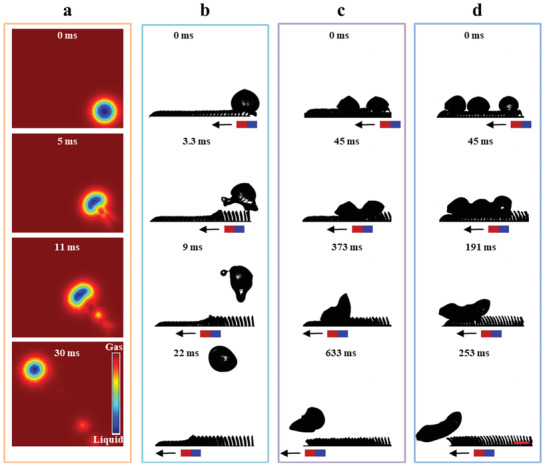
Cold droplets jumping dynamics on SMBA under magnetic field actuation. a) Simulation process of droplet pancake jumping on SMBA at *v*_m_ ≈ 150 mm s^–1^ by the Level‐set method. The simulated process of droplet pancake jumping is in accordance with the experimental observation. b) Selected snapshots showing that the cold droplet (≈ 0 °C) jumps away from SMBA at 3.3 ms when *v*_m_ ≈ 150 mm s^–1^. c) Two cold droplets (≈ 0 °C) can coalesce into one irregular droplet and detach from SMBA easily at 633 ms when *v*_m_ ≈ 10 mm s^–1^. d) Selected snapshots showing that three cold droplets (≈ 0 °C) coalesce into one droplet and rapid shed from SMBA at 253 ms when *v*_m_ ≈ 10 mm s^–1^. Scale bar: 2 mm.

To identify the key surface structural parameters and magnetic field features for the occurrence of pancake jumping, we plotted the variation of *k* as a function of the magnet moving velocity *v*_m_ on SMBA featuring with various aspect ratios *β* (**Figure** **4**). Apparently, the behavior of droplet motion is closely dependent on *β* and *v*_m_. On the surface with *β* ≈ 5, when the *v*_m_ ranges from 10 to 150 mm s^–1^, the increase of droplet liquid‐vapor surface area can be ignored (*k* ≈ 1), and thus, the kinetic energy gained from the interfacial energy is insufficient to propel the droplet to jump and droplet can only roll on the surface (Figure S13a, Supporting Information). Instead, an increase in *v*_m_ or *β* results in a significant increase in kinetic energy, and the droplet can jump away from the surface (on the surface with *β* ≈ 8 at *v*_m_ > 30 mm s^–1^, Figure S13b, Supporting Information, as well as on the surface with *β* ≈ 11 at *v*_m_ < 90 mm s^–1^). Note that on the surface with *β* ≈ 11 at *v*_m_ ≥ 90 mm s^–1^ (*k* ≥ 1.8), the droplet exhibits pancake jumping behavior. Interestingly, the occurrence of pancake jumping can be observed on SMBA even when *v*_m_ is ≈ 10 mm s^–1^ (*k* ≈ 2.1), further illustrating that the onset of pancake jumping necessitates the proper control of *β* and *v*_m_. We also simulated droplet pancake jumping dynamics using the Level‐set method (Figure S6, Supporting information) and the simulated results match well with the experimental observation (**Figure** **5**a).

## 3 Discussion

Icing accumulation, including frosting, condensate droplet freezing, and frozen rain, causes a series of damage in daily life, such as on the aircraft, power lines, and refrigeration system. Among various anti‐icing conditions, cold droplets are most difficult to remove. However, the traditional icephobic surface is usually limited by the high adhesion energy of the ice‐solid interface.^[^ ^1^, ^2^ ^]^ Considering the rapid droplet detachment from the substrate under magnetic field actuation,^[^ ^17^, ^38^, ^39^, ^40^, ^41^, ^42^, ^43^ ^]^ we further exploit the pancake jumping on SMBA for anti‐icing application. As present in Figure 5b, a cold droplet in an ice‐water mixed state (≈ 0 °C) deposited on SMBA can jump off the surface at *v*_m_ ≈ 150 mm s^–1^ (Figure S14, Supporting Information). The adhesion energy between the cold droplet and SMBA is far lower than the interfacial energy. So the cold droplet can easily detach from SMBA at 3.3 ms. The interaction time of the cold droplet with SMBA is slightly larger than that of the sessile water droplet at room temperature, due to the increased liquid viscosity of the cold droplet. Moreover, multiple cold droplets can also be rapidly shed off SMBA under magnetic field actuation. For example, when the magnet moves horizontally with *v*_m_ ≈ 10 mm s^–1^, multiple cold droplets sitting on SMBA can coalesce into an irregular droplet in an ice‐water mixture state. The irregular droplet moves forward and finally detaches from the surface (Figure 5c,d). The easy removal of cold droplets facilitated by the bending‐to‐straight response behavior of the blades array on SMBA is promising for anti‐icing application.

## 4 Conclusion

In summary, we discover a new phenomenon characterized by the jumping of sessile water droplets in a pancake shape. The pancake jumping is triggered by the fast response and quick deformation of superhydrophobic magnetic blades array and shows signatures of short interaction time and high energy conversion efficiency compared with other droplet bouncing behaviors. The boundary conditions for the occurrence of pancake jumping were theoretically simulated and experimentally validated. We also demonstrate the fast shedding of cold water droplets for anti‐icing applications. We envision that the discovery of droplet pancake jumping not only enriches and extends our understanding of dynamic liquid/solid interaction, especially involving external stimuli, but also offers the potential for a wide range of applications in droplet manipulation, microfluidic and micro‐robotic.^[^ ^1^, ^2^, ^3^, ^4^, ^5^, ^6^, ^7^, ^48^, ^49^ ^]^

## 5 Experimental Section

### Fabrication of PMMA Master Mold

The PMMA mold (thickness = 3 mm) patterned with rectangular holes array was created using a commercial CO_2_ laser‐engraving system (PLS6MW, Universal Laser Systems, Inc., Scottsdale, AZ; 75 W, laser wavelength = 10.6 µm). The laser beam was raster‐scanned across the surface at an output laser power of 15 W, with a speed of 0.254 m s^–1^ and a frequency of 30 kHz. The varied aspect ratios of SMBA can be obtained based on the structural parameters of rectangular hole arrays on the PMMA master mold. The width of the rectangular holes array can be adjusted in the vector graphics illustration software (Corel DRAW) before printing it in the laser scanning system. The depth of the rectangular hole arrays can be controlled by the optimized laser scanning power and laser scanning velocity.

### Fabrication of SMBA

Pure polydimethylsiloxane (PDMS) prepolymer (Sylgard 184, Dow Corning, USA) and curing agent were first mixed in a mass ratio of 15:1. Then carbonyl iron powders (CIP) (3.9–5.0 µm particle size, Fe>99.5%, SQ, BASF, Germany) in a weight ratio of 70% were added and thoroughly mixed with the PDMS solution. The PDMS/iron particle solution was cast into the prepared PMMA mold and degassed in a vacuum chamber for 1 h to allow the solution to completely occupy the rectangle holes on the mold, which was modified with 1H,1H,2H,2H‐perfluorooctyl trichlorosilane (Sigma Aldrich, USA) by chemical vapor deposition (CVD) for 1 h under vacuum in advance to reduce the adhesion strength between the mold and PDMS elastomer. To attain a strong magnetic response, an external magnetic field from a square permanent neodymium‐iron‐boron (NdFeB) magnet (45×45×20 mm) was placed underneath the PMMA mold for 10 min. The magnet can guide the magnetic microparticles into the holes. Extra PDMS/iron particle solution outside the holes was gently removed using a razor blade. After that, a pure PDMS (weight ratio 10:1 between the prepolymer and curing agent) solution was poured onto the PMMA mold and degassed for 0.5 h to remove the air bubble existing in the PDMS solution. After curing at 80 °C for 4 h, the sample with a magnetic blades array was obtained by peeling off the mold carefully. Furthermore, to render superhydrophobicity of the responsive surface, the laser raster‐scanning process was employed to gently ablate the superficial layer of the surface at a laser power of 20 W, laser velocity of 2.54 m s^–1^, and laser frequency of 30 kHz.

### Characterization of SMBA

The morphologies of the SMBA were observed by S‐4800 field emission scanning electron microscope (S‐4800 II FESEM, Hitachi High‐Technologies Corporation, Japan) with a high vacuum mode and an accelerating voltage of 15 kV. The static water contact angle, contact angle hysteresis, and sliding angle on SMBA were measured with a Ramé‐Hart goniometer (model 290‐U1). Droplets of ≈10 µL volume were gently deposited on the samples, the contact angles were measured using the optical fiber goniometer. To ensure repeatability of the results, all experiments were repeated three times at different locations on each sample. The magnetic flux density was measured with a digital Gauss meter (TD8620, Tunkia Co., Ltd. China).

### Droplet Pancake Jumping Experiment

The experiment was performed in an ambient environment, at room temperature 20 °C with 50% relative humidity. The experimental platform included two jointed NdFeB permeant magnets of the same sizes (45×45×20 mm), a horizontal sliding rail system, and a high‐speed camera (Phantom v1212, Vision Research, USA). The horizontal sliding rail system was composed of a horizontal single‐axis sliding table with a 28 linear stepper motor inside, a stepper motor single‐axis controller, a 28 stepper motor driver, and a DC power supply. The two jointed NdFeB permeant magnets, which were mounted on the sliding table, can horizontally move at 0 ∼ 150 mm s^–1^ by adjusting pulse frequency through the stepper motor single‐axis controller. The magnetic flux density was constant in all experiments (≈285 mT at the base of the blades array), owing to the fixed vertical distance between the blade array and the magnets. To generate a strong external magnetic actuation, the sample is placed right above the junction of two square NdFeB permeant magnets, where the deflected blades are in the maximum bending angle and form an end‐to‐end continuous surface. To avoid the movement of the magnet changing the position of the sample, the distance between the sample and the magnet is ≈ 2 mm. Then, a deionized water droplet with a radius *R* of ≈ 1.3 mm was deposited on the surface. When the magnets move away from the surface horizontally, the blades recover from the bending state to the straight state, resulting in the droplet that sits on the bending blades with different behaviors. The dynamic process of droplet behavior was recorded by using the high‐speed camera at 5000 frames per second from the side view. The cold droplet was generated by freezing a 10 µL denoized sessile water droplet on a cooling stage (IC20 C‐P, Cole‐Parmer Instrument Company, USA) at −10 °C. Then the cold droplet was transferred on SMBA. The shedding process of the cold droplet on SMBA was recorded by using a thermal imaging camera at 200 frames per second from the side view (FlLR A615, FLIR Systems, USA).

### Droplet Impact Experiment

The whole experiment was performed in an ambient environment, at room temperature of 20 °C with 50% relative humidity. The droplet with a radius of ≈ 1.3 mm formed at the tip of a steel needle from a syringe pump (LSPO1‐1A, LongerPump, China). The dynamic process of the droplet impingement on SMBA was captured by a high‐speed camera (Phantom v1212, Vision Research, USA) at 10 000 frames per second from the side view.

### Coalescence‐Induced Droplet Jumping Experiment

The experiment was conducted on a Cu superhydrophobic surface with a static contact angle of 160°. The sample sealed inside a PMMA chamber was placed on the surface of a cooling stage (IC20 C‐P, Cole‐Parmer Instrument Company, USA). The temperature and the relative humidity of the PMMA chamber were 20 °C and 70%, respectively. The temperature of the sample was kept at −5°C during the experiment. The dynamic process of coalescence‐induced droplet jumping was recorded by using a high‐speed camera (Phantom v1212, Vision Research, USA) at 1000 frames per second from the side view.

### Statistical Analysis

The droplet dynamics were analyzed with Phantom v1212 (Vision Research, USA) and Image J (National Institutes of Health, USA) software. Statistical analysis was done by using Origin 8.0 (Origin‐Lab Corporation, USA) software and all results were reported as means±standard deviation. In all the statistical analyses, the differences between multiple groups were analyzed using the one‐way analysis of variance (ANOVA), and the values of *p* < 0.05 were considered as statistically significant.

## Conflict of Interest

The authors declare no conflict of interest.

## Author Contributions

X.C., Q.L., and Z.W. conceived and supervised the research. X.C., C.Q., and Z.W. designed the experiments, C.Q. prepared the samples and carried out the experiments, C.Q., X.C., and Z.W. analyzed the data, F.Z. and D.H. ran the simulations. X.C., C.Q., F.Z., T.W., Q.L., and Z.W. wrote the paper. All authors discussed the results, proofread the paper, made comments, and approved the manuscript.

## Supporting information

Supporting Information

Supplemental Movie 1

Supplemental Movie 2

Supplemental Movie 3

Supplemental Movie 4

## Data Availability

The data that support the findings of this study are available from the corresponding author upon reasonable request.
