# Supplementary material for: Pancake Jumping of Sessile Droplets
Source: Adv Sci (Weinh). 2022 Jan 14;9(7):2103834. doi: 10.1002/advs.202103834 (PMC8895051; doi:10.1002/advs.202103834)
Supplement: Supplementary file 1 — Supporting Information [file ADVS-9-2103834-s004.pdf]

© 2020 Wiley-VCH GmbH

ADVANCED SCIENCE
Open Access

Supporting Information

for *Adv. Sci.,* DOI: 10.1002/advs.202103834

Pancake Jumping of Sessile Droplets

Chenlu Qian, Fan Zhou, Ting Wang, Qiang Li, Dinghua Hu, Xuemei Chen*, and Zuankai Wang**

Supporting Information

Pancake jumping of sessile droplets

Chenlu Qian[1], Fan Zhou[1], Ting Wang[2], Qiang Li[1*], Dinghua Hu[1], Xuemei Chen[1*], Zuankai Wang[2*]

[1]MIIT Key Laboratory of Thermal Control of Electronic Equipment, School of Energy and Power Engineering, Nanjing University of Science and Technology, Nanjing 210094, China.

[2]Department of Mechanical Engineering, City University of Hong Kong, Hong Kong 999077, China.

*Correspondence and requests for materials should be addressed to Q.L. (email: liqiang@njust.edu.cn) or to X.C. (email: xuemeichen@njust.edu.cn) or to Z. W.

(Email: zuanwang@cityu.edu.hk)

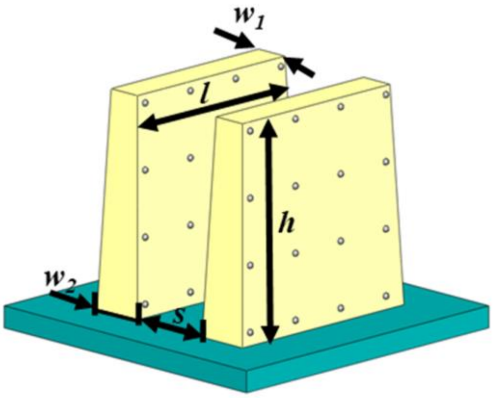

Figure S1. Schematic illustration of structures of SMBA. w_1 and w_2 is the blade width, h is the blade height, s is the spacing between two neighboring blades and l is the whole length of a blade.

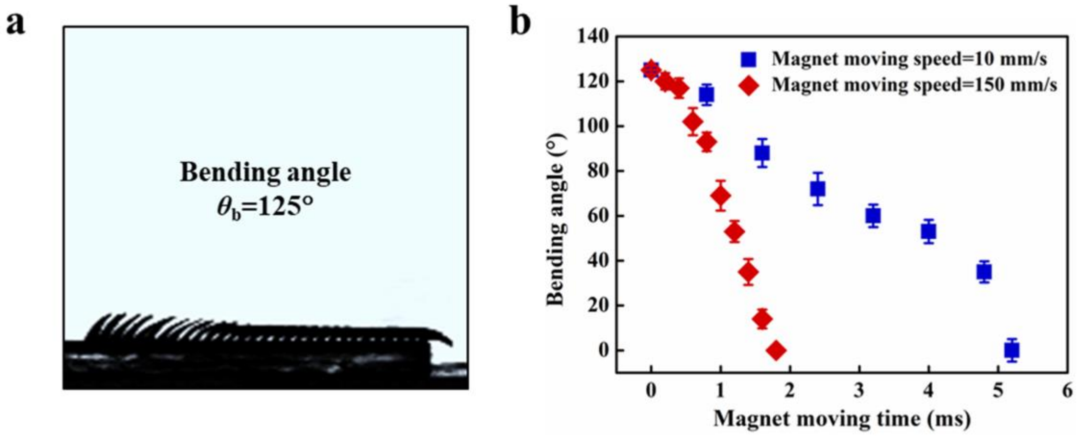

Figure S2. Maximum bending angle for SMBA under the magnetic field actuation (~ 285 mT). a) The experimental bending angle for SMBA is 125°. b) The bending-to-straight response time of single blade on SMBA is 5.2 ms at a magnet moving speed of 10 mm s^{-1}, and the bending-to-straight response time decreases to 1.8 ms at a magnet moving speed of 150 mm s^{-1}.

Table S1. Dimension parameter of the superhydrophobic magnetically-responsive blades array.

	w_1(μm)	w_2 (μm)	h (μm)	s (μm)	l (mm)	β
Aspect ratio of ~5	67	345	995	280	15	4.83
Aspect ratio of ~8	70	300	1450	215	15	7.84
Aspect ratio of ~11	44	287	1819	235	15	11.02
Aspect ratio of ~14	40	200	1675	280	15	13.96

Table S2. Experimental conditions of droplet pancake jumping and bouncing behaviors on the SMBA surface (corresponding to Figure 2 and Figure S8).

	Magnet moving speed (mm s^{-1})	Weber number	Maximum jumping/bouncing height (mm)
Pancake jumping	150	0	11.4
Oblique bouncing	150	5	6.4
Conventional bouncing	0	10	3.0
Pancake bouncing	without magnet field action	34	2.2

Sections

Section 1: The comparison of practicability between pancake jumping in this work and pancake bouncing or droplet jumping in a lower pressure environment.

1. Comparison between pancake jumping and pancake bouncing.

As can be seen in Liu et al, *Nat Phys*. 2014,10,515,[1] pancake bouncing occurs on straight posts with $1.0 < k < 1.7$ when *We* in a restricted range or occurs on the tapered surface with $0.5 < k < 1.7$ when $8.0 < We < 58.5$. k is a dimensionless number related to the surface structural parameters. The emergence of pancake bouncing needs to design optimized structures and control the limited droplet impacting velocity.

As can be seen in Figure 4, the droplet pancake jumping occurs on SMBA with $ß \sim 11$ when $v_m \geq 90$ mm s^{-1} or on SMBA with $ß \sim 14$ when $v_m \geq 10$ mm s^{-1}. The occurrence of pancake jumping only needs to control the aspect ratio of the blades array. Through the combined soft lithography and laser ablation technology, the magnetic blades array can be easily fabricated. The varied aspect ratios *ß* of SMBA can be obtained based on the structural parameters of rectangular hole arrays on the PMMA master mold. The width of the rectangular holes array can be adjusted in the vector graphics illustration before printing it in the laser scanning system. The depth of the rectangular holes array can be controlled by the optimized laser scanning power and laser scanning velocity.

2. Comparison between pancake jumping and spontaneous droplet trampolining.

As shown in Schutzis et al, *Nat*. 2015, 572, 82,[2] water droplets resting on the superhydrophobic textured surfaces in a low-pressure environment can trampoline

spontaneously. The environment pressure (0.01 bar) needs to be accurately controlled through a complicated environmental chamber. The environmental chamber consists of an aluminum-based pressure vessel connected to a vacuum pump, a pressure sensor and a pressurized nitrogen reservoir. The low humidity is controlled by nitrogen flow and the low pressure is adjusted by the pressure pump in the chamber.

However, droplet manipulation under an ambient environment is more common in practical applications. In particular, owing to its advantages of instantaneous response,[3] low energy consumption, flexible/convenient/safe controllability and good biocompatibility, magnetic actuation has emerged as a promising approach to manipulate droplet motion.[4] In our experiment, the emergence of droplet pancake jumping can be easily realized in the ambient pressure and humidity. When the droplet is deposited on the SMBA, the droplet motion can be controlled by moving the magnet. The magnet is mounted on a horizontal sliding rail system, which was composed of a single-axis sliding table with a 28 linear stepper motor inside. The magnet moving velocity is adjusted by changing the pulse frequency of the stepper motor.

Section 2: Numerical simulation of droplet pancake jumping.

We performed numerical simulation of droplet pancake jumping on the SMBA surface, which is divided into two parts: calculation the deflecting process of a blade under external magnetic field actuation using the coupled *Fluid-Structure interaction, Magnetic Fields* (*CFSIMF*) method, and simulation of the dynamic process of droplet pancake jumping with the *Level-set* (*LS*) method. The induced magnetic flux density of the blade $\overrightarrow{B_m}$ under external magnetic field is given by

$$\overrightarrow{B_m} = \mu_0\,\mu_r\,\vec{H} \tag{S1}$$

where μ_0 is the permeability of vacuum, μ_r is the relative permeability (μ_r = 3) and H is the external magnetic field strength. The magnetic force $\overrightarrow{F_m}$ acting on the blade is calculated as an integral of the surface stress tensor over all boundaries of the blade, and the stress tensor is expressed as

$$\vec{n}T = \frac{1}{2}\left(\vec{H}\cdot\overrightarrow{B_m}\right)\vec{n} + \left(\vec{n}\cdot\vec{H}\right)\overrightarrow{B_m}^T \tag{S2}$$

where $\vec{n}$ is the boundary normal pointing out from the blade and T is the stress tensor of air.

Meantime, the air flow is described by the incompressible Navier-Stokes Equations for the velocity field and the pressure in the spatial (deformed) moving coordinate system:

$$\rho_i \frac{\partial \overrightarrow{u_i}}{\partial \mathrm{t}} - \nabla\cdot\left[-p\vec{I} + \mu(\nabla\overrightarrow{u_i} + (\nabla\overrightarrow{u_i})^T)\right] + \rho_i\left((\overrightarrow{u_i})\cdot\nabla\right)\overrightarrow{u_i} = \overrightarrow{F_m} \tag{S3}$$

$$-\nabla\cdot\overrightarrow{u_i} = 0 \tag{S4}$$

where ρ_i is the density of air, $\overrightarrow{u_i}$ is the air velocity field, p is the pressure, $\vec{I}$ denotes the unit diagonal matrix and μ is the dynamic viscosity of air. According to the second Piola-Kirchhoff stress, equation of the blade motion can be expressed as

$$\rho_{PDMS}\frac{\partial^2\overrightarrow{u_s}}{\partial t^2} = \nabla\cdot\left(\overrightarrow{F_{grd}}S\right)^T + \overrightarrow{F_V} \tag{S5}$$

where ρ_{PDMS} is the density of PDMS (970 kg m^{-3}), $\overrightarrow{u_s}$ is the solid velocity field, $\overrightarrow{F_{grd}} = \vec{I} + \nabla\overrightarrow{u_s}$ is the deformation gradient, $\overrightarrow{F_V} = \frac{\overrightarrow{F_m} - \vec{n}[-p\vec{I} + \mu(\nabla\overrightarrow{u_l} + (\nabla\overrightarrow{u_l})^T)]}{V_{PDMS}}$ is the volume force vector, and S is the total stress. The total stress is written as $S = S_0 + \vec{C}:\epsilon$, where S_0 is the initial stress, $\vec{C} = \vec{C}(E, \sigma)$ is the right Cauchy-Green deformation tensor (Poisson's ratio σ=0.49) and $\epsilon = \frac{1}{2}[(\nabla\overrightarrow{u_s})^T + \nabla\overrightarrow{u_s} + (\nabla\overrightarrow{u_s})^T\nabla\overrightarrow{u_s})$ is strain represented by the Green-Lagrange strain tensor. Combined Eq. 3-5, bending angle of the blade with 8471, 15167, 23913, 31643 and 55971 grids are calculated as 32.88°, 33.62°, 33.67°, 33.75° and 33.74° respectively (Supplementary Figure 3). Considering the calculation costs, we use the case with 31643 in the rest simulations, in which the 'normal' grid was selected in the COMSOL.

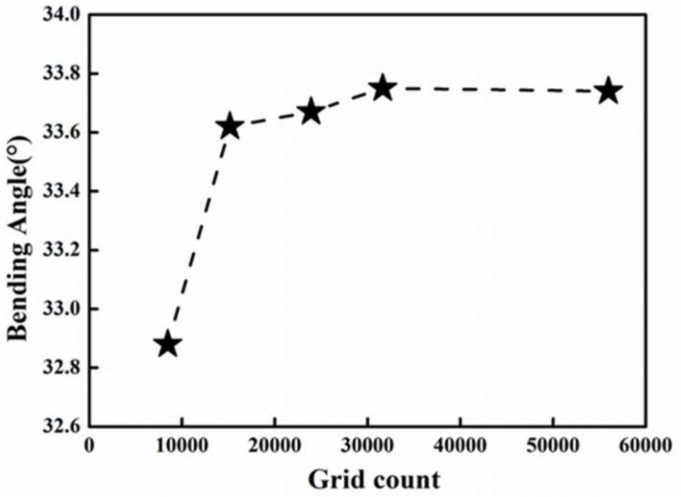

Figure S3. Grid independence analysis. The 31643 grids is used in the simulation.

The magnetic field of two jointed square NdFeB permeant magnets (45×45×20 mm) is simulated and the magnetic force of a blade with aspect ratio of ~14 is analyzed. The direction of the magnetic force acting on the blade points to the gradient of external magnetic field strength and the magnitude of magnetic force is determined by the position of the blade in the magnetic field, whose horizontal component and vertical component is symmetrical about the

center of the magnet (Figure S4). The simulated bending angle $\theta_{simulation}$ of the single blade, which related to the magnetic force and elastic force, is up to 124.5° with elastic force 0.009146 N under the external magnetic actuation (Figure S4c).

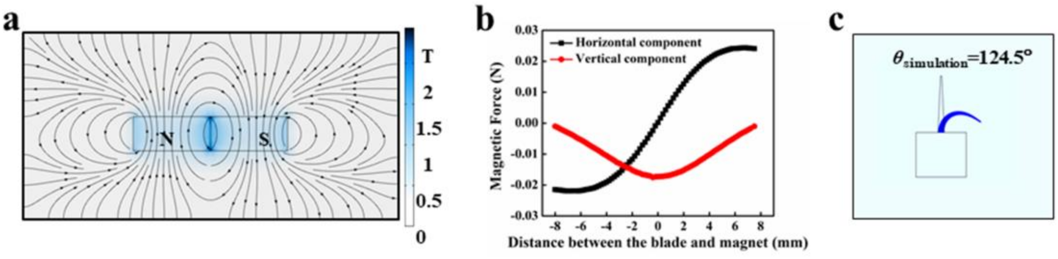

Figure S4. Simulation of magnetic force exerted on a blade with an aspect ratio of ~ 14. a) Simulation of the external magnetic field. b) The horizontal component and vertical component of the magnetic force is symmetrical about the center of the magnet. c) The simulated maximum bending angle for SMBA is 124.5°.

The elastic force is equal to the magnetic force under the steady-state condition, inducing elastic hysteresis under the transient condition as comparison with the magnetic force changing immediately by the moving magnet. The magnetic force and the elastic force are calculated based on the bending deformation of one single blade (Figure S5). The magnitude of interfacial force acting on the droplet can be calculated by the difference of the magnetic force and the elastic force under the transient condition. The force point of magnetic force and the elastic force is on the top of the blade, but the force point of interfacial force is in the middle of the blade. According to the law of energy conservation, the actual magnitude of the interfacial force on one single blade is two times of the difference between the magnetic force and the elastic force under the transient condition. According to the experimental observation, the droplet pancake jumping is pushed by the rightmost four blades of SMBA. Therefore, the

simulated magnitude of interfacial force is calculated by the interfacial force of one blade multiplying by eight times.

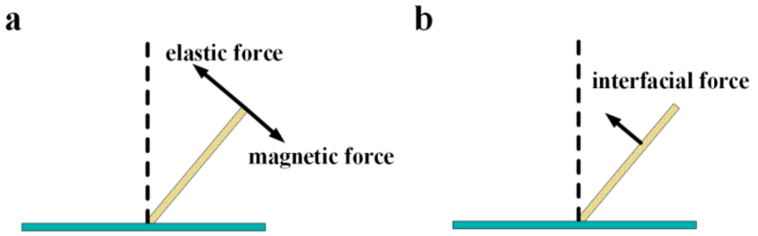

Figure S5. Schematic diagram of force analysis. a) Schematic diagram of the magnetic force and the elastic force acting on the single blade. b) Schematic diagram of the interfacial force acting on the single blade.

Taking the phase interface φ of 0.5, the droplet jumping height h_0 is calculated (Figure S6). The agreement between the numerical simulation and the experimental data suggests that the simulation well captures the essentials of the droplet pancake jumping process. The droplet pancake jumping dynamics on SMBA is also simulated by the *Level-set* (*LS*) method and is shown in Figure 6a, which matches well with the experimental observation.

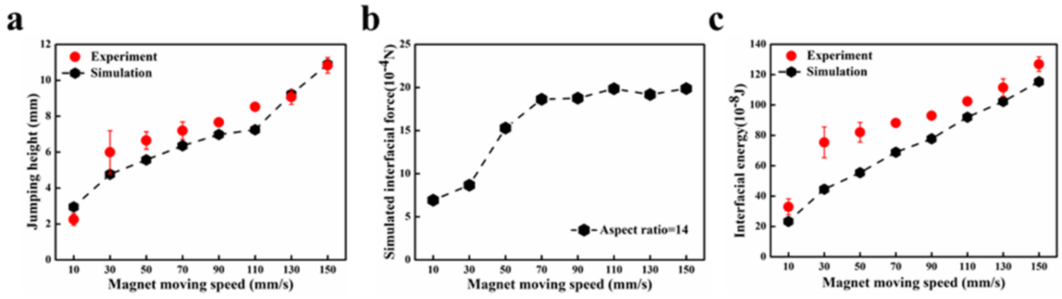

Figure S6. Simulation of the dynamic process of droplet pancake jumping on SMBA with the magnet moving speed of 150 mm s^{-1}.

Section 3: Comparison of droplet dynamic in this work and droplet jumping or bouncing behaviors in the literatures.

1. Comparison of droplet dynamics on SMBA and in a low-pressure environment[5] or on a heated substrate[6] or on a vibrating substrate.[7]

Similarity: The sessile droplets can all be impelled to jump off the surface by the external energy stimuli.

Differences: First, the external field stimulus for droplet jumping in a low-pressure environment or on a heated substrate or on a vibrating substrate are the environment pressure, surface temperature and vibration of the substrate via an adjustable electromagnet respectively; whereas the droplet dynamics on SMBA is subjected to the bending deformation of the blades array under magnetic field actuation. Second, the droplets in a low-pressure environment or on a heated substrate or on the rigid vibrating superhydrophobic plate are propelled to move upward under a vertical upward force; whereas the droplet on SMBA obliquely jumps off the surface under a vector force (Figure 2a). Third, the droplet on the rigid vibrating surface undergoes flattened deformation before leaving the plate; whereas the droplet on SMBA forms an arc shape before jumping off the surface and processes a pancake shape in the air. Fourth, the droplet motion on the rigid vibrating superhydrophobic plate needs to subtlety match the frequency of droplet motion and the vibration of the plate; the droplet trampolining in a low-pressure environment need to control the environment pressure accurately; whereas the pancake jumping for the droplet on SMBA is dependent on the aspect ratio of the blade and the magnet moving speed.

2. Comparison of droplet pancake jumping on SMBA and droplet impacting on a

superhydrophobic flexible surface.[8]

For the droplet impacting on the flexible superhydrophobic surface, there exist two energy conversion mechanisms: kinetic-to-interfacial energy within the droplet and kinetic-to-elastic energy between the droplet and the elastic substrate. At the early stage of impact, the elasticity of the substrate enables the conversion of kinetic energy to elastic energy, thus no sufficient kinetic energy is left for kinetic-to-interfacial energy conversion. Besides, an upward force from the oscillating substrate causes the droplet to detach before fully undergoing interfacial-to-kinetic energy conversion.

For pancake jumping on SMBA, there exist three energy conversion mechanisms: magnetic/elastic-to-rotation energy within the blades array; rotation-to-interfacial energy between the blades array and the droplet; interfacial-to-kinetic energy within the droplet. Based on the energy balance, the rotation energy is completely converted into the droplet interfacial energy. The energy conversion efficiency of pancake jumping (~ 95%) is 23 times larger than that of droplet bouncing off the flexible surface (~ 4%), as can be seen in Table S3.

Table S3. Comparison of energy conversion efficiency between the pancake jumping in this work and bouncing behaviors in the literatures.

Droplet motion behaviors	Energy conversion efficiency	Reference
Pancake jumping in this work	95%	This work
Bouncing off the flexible superhydrophobic surface	4%	8
Coalescence-induced jumping	20%	9
Conventional bouncing	13%	10
Pancake bouncing	8%	11

Bouncing on slippery liquid interfaces	81%	12
Droplet oblique bouncing on the inclined superhydrophobic surface	65%	13
Hot droplet bouncing	9%	14

Section 4: The influence of surface edge effect on droplet dynamics on SMBA.

For the blades array on SMBA under magnetic actuation, only the rightmost one can reach its maximum bending angle (~ 125°). The blades in the center of SMBA are subjected to a neighborhood edge effect, which causes the blades to reach a bending angle of only ~ 90°. For pancake jumping (Figure 2a in the manuscript), the sessile droplet is initially deposited on the rightmost of SMBA. The droplet can jump off the surface in a pancake shape because off the sufficient kinetic energy gained from the bending-to-straight process of the blades on the rightmost of the surface. However, when the droplet is initially deposited in the center of SMBA, the droplet exhibits conventional jumping (Figure S7a). For oblique bouncing, when the droplet impacts on the rightmost of SMBA, the droplet bounces off the surface obliquely due to enough kinetic energy provided by the bending blades deformation (Figure 2b in the manuscript). However, when the droplet impacts on the center of SMBA, the droplet exhibits conventional bouncing (Figure S7b).

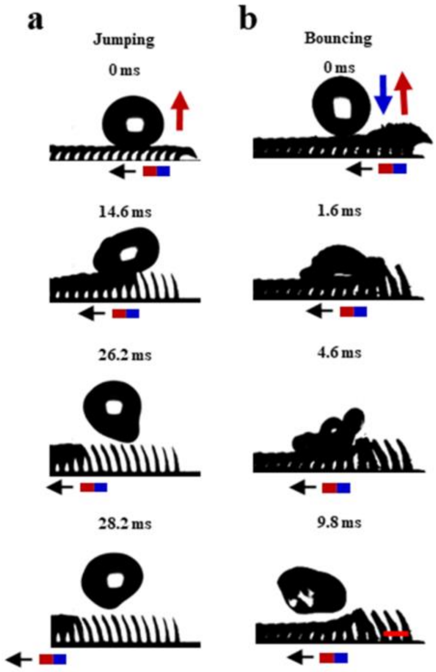

Figure S7. Droplet motion dynamics. a) Droplet exhibits conventional jumping at v_m ~ 150 mm s^{-1} when it is deposited in the center of SMBA. b) Droplet exhibits conventional bouncing at v_m ~ 150 mm s^{-1} when it impacts on the center of SMBA. Scale bar: 1 mm.

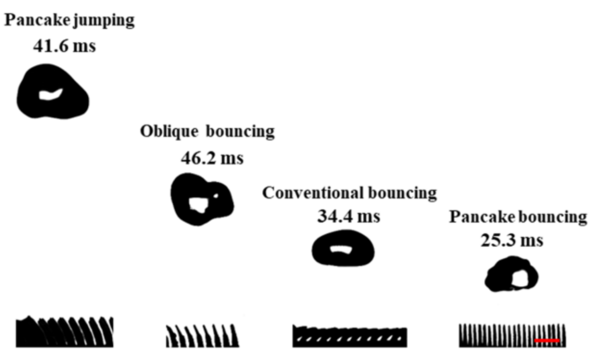

Figure S8. Selected snapshots showing the maximum jumping height of the pancake jumping, oblique bouncing (initial impacting height ~ 6.3 mm), conventional bouncing (initial impacting height ~ 11.5 mm) and pancake bouncing (initial impacting height ~ 40 mm). Scale bar: 1 mm.

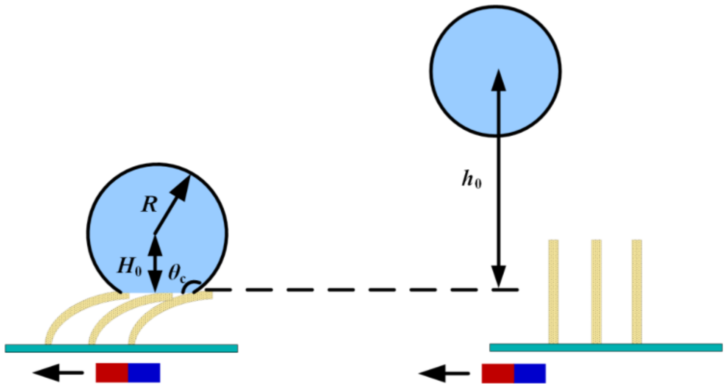

Figure S9. Schematic diagram of initial height H_0 and maximum jumping height h_0 of pancake jumping. h_0 is the maximum jumping height between the droplet centroid and the surface. H_0 is the initial height of the droplet between the gravitational center of the droplet and the surface in the initial state.

Section 5: Mechanism of droplet pancake jumping.

Droplet pancake jumping can be divided into two periods: droplet deforming when it contacts with SMBA; droplet jumping in the air when it is not in contact with SMBA. When the droplet is in contact with SMBA, the energy balance is applied between the rotation energy of the blades array and the droplet interfacial energy. When the droplet jumps off the surface, the energy balance is applied between the droplet interfacial energy and the droplet kinetic energy.

The mechanism of pancake jumping is analyzed based on the change in interfacial energy of the droplet. When the bending blades array recovers to the straight state under a horizontal motion of the magnet, the unbalance of magnetic torque and elastic torque induces rotation energy of the blades array. Taken the droplet and the blades array underneath as a system, the rotation energy is completely transformed into the droplet interfacial energy. The droplet can form an arc shape because of the increase in droplet interfacial energy provided by the bending-to-straight deformation of the blades array.

Figure S10 schematically illustrates the droplet deformation when the bending blades array recovers to the straight state (corresponding to droplet dynamics from 0 ms to 1.8 ms in Figure 2a). Assuming that a spherical-cap droplet with radius R, height H and static contact angle θ_c placed on the deflected blades, the droplet liquid-vapor surface areas is A_{lv} ($A_{lv} = 2\pi R^2(1 - cos\theta_c)$) and the solid-liquid surface area is A_{sl}. When the deflected blades array recovers to the straight state under magnetic field actuation, the droplet forms an arc shape and its shape can be equivalent to two intersection segments. The droplet liquid-vapor surface areas increases to A_{lv}' and the droplet interfacial energy E_s generated from the blades

can be expressed as:

$$E_s = \gamma A_{lv}' - \gamma(A_{lv} - A_{sl}cos\theta_c) = \gamma A_{lv}' - \pi\gamma R^2(2 - 3\cos\theta_c + \cos^3\theta_c) \quad \text{(S6)}$$

where γ is the liquid surface tension.

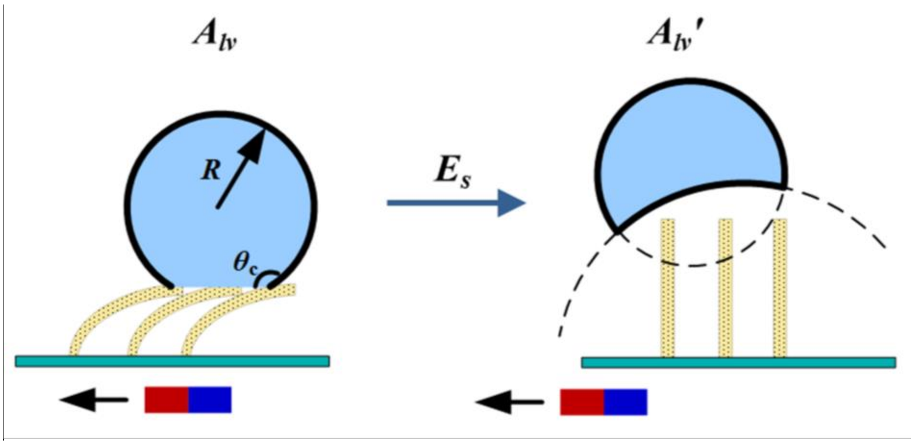

Figure S10. Schematic diagram of droplet deformation during the bending-to-straight response process of blades arrays under magnetic field actuation (corresponding to droplet dynamics from 0 ms to 1.8 ms in Figure 2a). A spherical-cap droplet is deposited on SMBA, the droplet liquid-vapor surface area is $A_{lv.}$ When the blades array recovers from the bending state to the straight state, the liquid-vapor surface area of the droplet increases to A_{lv}', in which the droplet interfacial energy is generated from the blades array under magnetic field actuation.

The energy of the deflected blade is analyzed when it recovers from the bending to the straight state under the magnetic field actuation (Figure S11). The elastic torque T_e which is dependent on the bending angle θ_b can be expressed as:

$$T_e = K_{eq}\theta_b \quad \text{(S7)}$$

where K_{eq} is the equivalent torsion spring constant, determined by the elastic modulus of a blade ($E\approx$1.33 MPa), the second moment of inertia I ($I = \frac{l}{12}(\frac{w_1+w_2}{2})^3$), the blade length l, the

blade top width w_1, bottom width w_2, and a parametric angle coefficient c. K_{eq} is given as:

$$K_{eq} = \frac{cEI}{h} \tag{S8}$$

and the elastic torque T_e is expressed as:

$$T_e = \frac{cEI}{h}\theta_b \tag{S9}$$

Meanwhile, carbonyl iron particles inside a magnetic blade experience a magnetic field torque in an external magnetic field. The magnetic torque T_m which rotates the carbonyl iron particles into alignment with the magnetic field can be given as:

$$T_m = V_m \vec{M} \times \vec{B} \tag{S10}$$

Where V_m is the volume of the carbonyl iron particles inside a magnetic blade, M is the magnetization of the magnetic blade which is assumed to be in the axial direction and B is the magnetic flux density. The magnitude of this magnetic torque is

$$T_m = V_m MB \sin(\alpha_m - \theta_b) \tag{S11}$$

where (α_m-θ_b) is the angle between the external magnetic field and the bending magnetic blade and α_m is the magnetic field angle, which defined as the angle between the applied field and vertical direction (α_m = 0° at the junction of two NdFeB permanent magnets).

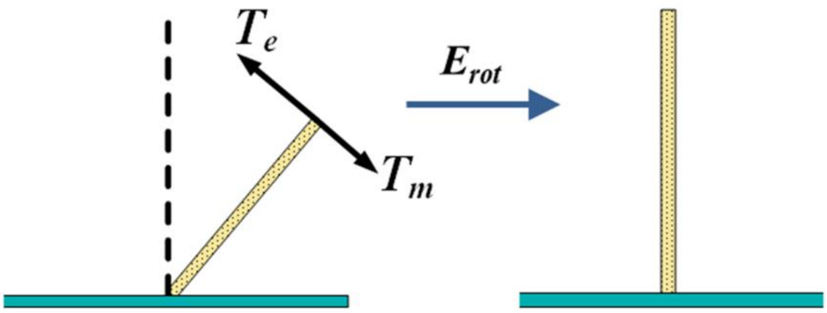

Figure S11. Energy analysis of a magnetically-responsive blade under magnetic field actuation. The blade is exerted on the elastic torque and magnetic torque when it recovers from the bending state to the straight state.

When the magnetic blade recovers from the bending state to the straight state at time interval t_m with the magnet moving speed of v_m, the rotation energy can be expressed as

$$E_{rot} = \int_{\theta_b}^{0} (T_e - T_m)\, d\theta \sim \frac{cEl(\frac{w_1+w_2}{2})^2}{24} \cdot \frac{{\theta_b}^2}{\beta} + \frac{V_m M B_0}{(v_m t_m)^3} \cdot |cos\theta_b| \quad \text{(S12)}$$

where β is the blade aspect ratio ($\beta = h/(\frac{w_1+w_2}{2})$), the magnetic flux density $B \sim \frac{B_0}{(v_m t_m)^3}$ and B_0 is the initial magnetic flux density (B_0 = 285 mT).

Based on the conservation of energy, the droplet interfacial energy of the blade is equal to the rotation energy of the blade. Combined Equation S6 and Equation S12, when the deflected blades array recovers to the straight state, the droplet liquid-vapor surface area A_{lv}' can be written as

$$A_{lv}' = \pi R^2 (2 - 3\cos\theta_c + \cos^3\theta_c) + \frac{cEl(\frac{w_1+w_2}{2})^2}{24\gamma} \cdot \frac{{\theta_b}^2}{\beta} + \frac{V_m M B_0}{\gamma (v_m t_m)^3} \cdot |cos\theta_b| \quad \text{(S13)}$$

By defined $f_1 = \frac{cEI}{\gamma(w_1+w_2)}$ and $f_2 = \frac{V_m M B_0}{\gamma}$, A_{lv}' is simplified as

$$A_{lv}' = \pi R^2 (2 - 3\cos\theta_c + \cos^3\theta_c) + f_1 \frac{{\theta_b}^2}{\beta} + f_2 \frac{|cos\theta_b|}{(v_m t_m)^3} \quad \text{(S14)}$$

Thus, k is defined as the ratio of droplet interfacial energy after and before the deflected blades array recovering to the straight state, and can be expressed as

$$k \sim \frac{{\theta_b}^2}{\beta} + \frac{|cos\theta_b|}{(v_m t_m)^3} + 1 \quad \text{(S15)}$$

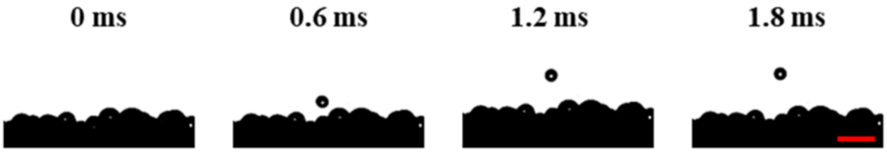

Figure S12. Coalescence-induced condensate droplet jumping on a Cu superhydrophobic surface. Scale bar: 1 mm.

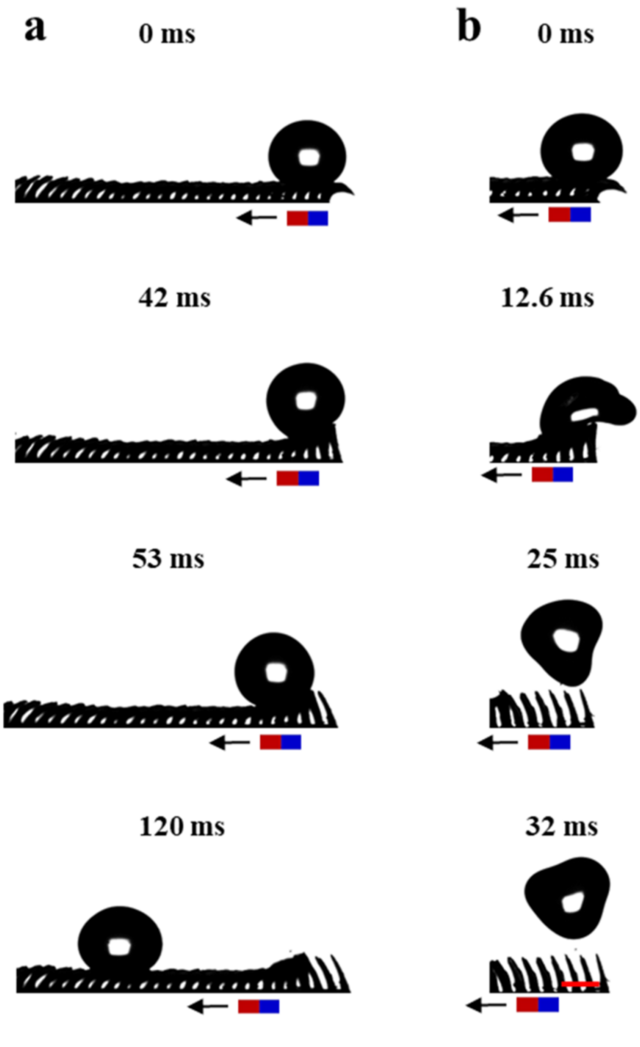

Figure S13. Different water droplet motion behaviors under magnetic field actuation on the superhydrophobic magnetically-responsive surface with aspect ratio of ~ 8. Selected snapshots showing the water droplet was propelled to a) roll on the surface with an aspect

ratio of ~ 8 at a magnet moving speed of 10 mm s^{-1} and b) jump away from the surface with an aspect ratio of ~ 8 at a magnet moving speed of 150 mm s^{-1}. Scale bar: 1 mm.

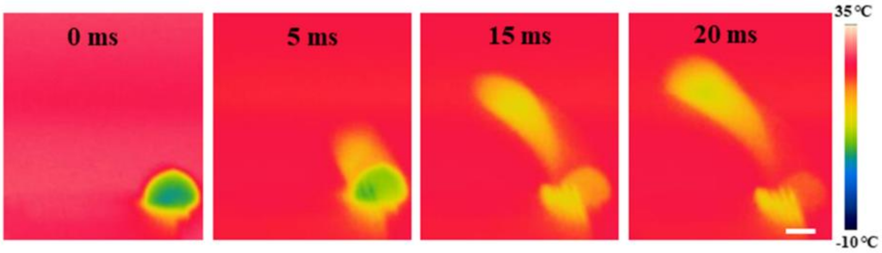

Figure S14. Selected snapshots showing a frozen droplet jumping off SMBA with v_m ~ 10 mm s^{-1} and captured by the thermal imaging camera. Scale bar: 2 mm.

Supporting Movies

Movie S1. Droplet pancake jumping off SMBA with the magnet moving speed of 150 mm s^{-1} is shown.

Movie S2. Droplet oblique bouncing off SMBA with the magnet moving speed of 150 mm s^{-1} is shown.

Movie S3. Droplet conventional bouncing off SMBA with the magnet stationary is shown.

Movie S4. Droplet pancake bouncing off SMBA without magnetic field actuation is shown.

Supporting References

[1] Y. Liu, L. Moevius, X. Xu, T. Qian, J. M. Yeomans, Z. Wang, *Nature Phys.* **2014**, *10*, 515.

[2] T. M. Schutzius, S. Jung, T. Maitra, G. Graeber, M. Köhme, D. Poulikakos, *Nature* **2015**, *527*, 82.

[3] A. Al-Azawi, M. Latikka, V. Jokinen, S. Franssila, R. H. A. Ras, *Small* **2017**, *13*, 1700860.

[4] S. Jiang, Y. Hu, H. Wu, Y. Zhang, Y. Zhang, Y. Wang, Y. Zhang, W. Zhu, J. Li, D. Wu, J. Chu, *Adv. Mater.* **2019**, *31*, 1807507.

[5] T. M. Schutzius, S. Jung, T. Maitra, G. Graeber, M. Köhme, D. Poulikakos, *Nature* **2015**, 527, 82.

[6] J. T. Pham, M. Paven, S. Wooh, T. Kajiya, H.-J. Butt, D. Vollmer, *Nat Commun.* **2017**, 8, 905.

[7] C. Raufaste, G. R. Chagas, T. Darmanin, C. Claudet, F. Guittard, F. Celestini, *Phys. Rev. Lett.* **2017**, *119*, 108001.

[8] P. B. Weisensee, J. Tian, N. Miljkovic, W. P. King, *Sci Rep.* **2016**, 6, 30328.

[9] J. B. Boreyko and C.-H. Chen, *Phys. Rev. Lett.* **2009**, *103*, 184501.

[10] J. C. Bird, R. Dhiman, H.-M. Kwon, and K. K. Varanasi, *Nature* **2013**, *503*, 7476.

[11] Y. Liu, L. Moevius, X. Xu, T. Qian, J. M. Yeomans, and Z. Wang, *Nat. Phys.* **2014**, *10*, 7.

[12] C. Hao, J. Li, Y. Liu, X. Zhou, Y. Liu, R. Liu, L. Che, W. Zhou, D. Sun, L. Li, L. X, Z. Wang, *Nat. Commun.* **2015**, *6*, 7986.

[13] D. G. K. Aboud, A.-M. Kietzig, *Langmuir* **2018**, *34*, 9889.

[14] T. Mouterde, P. Lecointre, G. Lehoucq, A. Checco, C. Clanet, D. Quéré, *Nat. Commun.* **2019**, 10, 1410
